# An opportunistic qualitative evaluation of the acceptability and feasibility of remote cognitive behavioral group intervention for bipolar disorder

**DOI:** 10.3389/fpsyg.2025.1418994

**Published:** 2025-11-07

**Authors:** Elizabeth Newton, Alex Copello, Alison Rolfe, Katie Edwards, Sarah Moonakova, Arielle Kaufman, Radha Yagnik, Olivia James, Anne Leadbitter, Gurvir Matharu

**Affiliations:** 1 Birmingham and Solihull Mental Health NHS Foundation Trust, Birmingham, United Kingdom; 2 School of Psychology, University of Birmingham, Birmingham, United Kingdom; 3 West Midlands Institute of Psychotherapy, Birmingham, United Kingdom; 4 School of Psychology, Aston University, Birmingham, United Kingdom

**Keywords:** bipolar disorder, CBT, remote delivery, online therapy, group intervention, psychoeducation, COVID-19, telemedicine

## Abstract

**Introduction:**

In response to the COVID-19 pandemic, many mental health services had to adapt the way in which their services were delivered. Research exploring the effectiveness of remote therapy and interventions, especially within the bipolar disorder population, is lacking. The pandemic presented an opportunity to conduct an opportunistic evaluation of a group CBT intervention for bipolar disorder which began face-to-face and transitioned to remote delivery. Service users had a unique experience of having experienced both delivery methods during the same intervention. The intervention had not previously been adapted or investigated in terms of online delivery and it was imperative to gain in-depth insight into service users and staff member’s experiences. The overarching aim of this evaluation was to provide qualitative insight into service users’ and staff members’ experiences of the feasibility and acceptability of online as compared and contrasted to face-to-face CBT intervention for bipolar disorder.

**Methods:**

A qualitative method was used to provide an in-depth, contextualized understanding of individual perceptions and experiences of two contrasting group delivery formats, face-to-face and remote. Individual interviews were undertaken with service users and a focus group was held with staff facilitating the group. Data were analyzed using thematic analysis.

**Results:**

The evaluation suggests that using video technology can be an effective way of delivering intervention to this client group and may have additional benefits such as easier access for some service users by reducing need to travel, easier access when struggling with mental health and aid in concentration when processing the group content. Collectively the analyses suggest that before embracing the use of technology for delivering psychological group interventions, we need to be cautious and consider clinical, group and practical processes that may be impacted and work towards diminishing these drawbacks. These factors and processes are discussed, including symptom management, accessibility, relationships and bonding, risk management and introducing a hybrid model.

**Discussion:**

This study provides initial support for the feasibility of delivering group CBT for bipolar disorder online and its acceptability. However, it also highlights some challenges and clinical considerations. Moving forward, services could consider offering service users a choice of either face-to-face or online delivery which may widen access to psychological interventions and promote inclusivity. Further research, both qualitative and quantitative, is needed within the bipolar population to explore remote delivery and help guide services when delivering online psychological interventions.

## 1 Introduction

Bipolar disorder (BD) falls under the larger diagnostic category of mood disorders. The onset of bipolar disorder usually occurs in adolescence and early adulthood (Jann, 2014). However, many people experience up to 10 years of symptoms before their diagnosis is identified (Fagiolini et al., 2013). Clemente et al. (2015) reviewed the literature and found a global lifetime prevalence of 1.1% for Bipolar I and 1.2% for Bipolar II. This varies greatly between countries, for example, systematic reviews in China and South Asia reported a lifetime prevalence of 0.11% (Zhang et al., 2017) and 0.6% (Naveed et al., 2020), respectively. In comparison, in the US the lifetime prevalence is estimated at 4.4% (Merikangas et al., 2011). Researchers have suggested similar rates of diagnosis across genders with slightly higher rates for females than males (Pini et al., 2005). Kawa et al. (2005) suggest that women were more likely to report mania at the onset of the disorder, whereas men were more likely to report depression at this stage.

The total burden of disease for mental illness in the UK is reported to be £117.9 billion a year with Bipolar Disorder accounting for 17% of this, compared to 23% for depression and 8% for schizophrenia (Bipolar Commission UK, 2022). Fajutrao et al. (2009) reviewed evidence from European countries and concluded that up to 75% of patients with a bipolar diagnosis have one or more DSM-5 comorbidities, with anxiety and substance abuse disorders being the most common. The World Health Organisation (WHO) state that, after neurological conditions, bipolar disorder has the second greatest effect on absenteeism from work compared to other mental and physical disorders and illnesses (Alonso et al., 2011; Grande et al., 2016).

Research indicates that between 25–60% of individuals with BD will attempt suicide at some point during their lives and between 4–19% will die by suicide (Novick et al., 2010). As such, the mortality rate of this condition is more than double that of the general population (Müller-Oerlinghausen et al., 2002). The increased risk, comorbidities and impact on day-to-day functioning in those with a diagnosis of bipolar disorder makes service provision essential.

The National Institute for Health and Care Excellence (NICE) recommends that individuals with a diagnosis of bipolar disorder should be offered a structured psychological intervention (individual, group or family) designed specifically for BD. This intervention should provide information about bipolar disorder, consider the impact of thoughts and behavior on moods, include self-monitoring of mood, address relapse risk and develop plans for relapse management/staying well and consider problem solving to address communication patterns and managing functional difficulties (National Institute for Health and Care Excellence, 2014). The NHS Long Term Plan and community transformation sets out to increase psychological interventions to individuals with severe and enduring mental health difficulties, including individuals with a diagnosis of bipolar disorder (National Health Service, 2019).

Chiang et al. (2017) conducted a meta-analysis of randomized controlled trials investigating the effectiveness of Cognitive Behavioral Therapy (CBT) for BD (both individual and group interventions). CBT was found to reduce relapse rates and improve depressive symptoms, mania severity and psychosocial functioning with a mild to moderate effect size. Group and individual CBT have also been reported to improve mood and social functioning, with treatment gains maintained up to 18 months post-intervention (Costa et al., 2010). Results also favored CBT plus medication over pharmacological treatment alone in terms of a reduction in relapse rates, hospitalization, impulsive behavior, frequency of mood crises and relationship conflicts. Group psychoeducation interventions for individuals with a diagnosis of BD have been found to reduce hospitalization rates (Buizza et al., 2019), involvement with crisis teams (National Institute for Health and Care Excellence, 2014) and delay manic relapses (Watson and Dodd, 2017).

In March 2020, the Coronavirus pandemic (COVID-19) induced a nationwide lockdown across the UK. Government guidance advised against the mixing of separate households, and recommended that, when meeting others was unavoidable, a two-meter distance should be maintained (Department of Health and Social Care, 2020). These rules drastically changed the way in which health services were allowed to operate with many services switching from face-to-face appointments to telephone or video consultations, enabling the provision of care to continue whilst complying with lockdown regulations. The imperative need to continue to provide therapeutic services, despite difficult circumstances, lay in the highly debilitating nature of BD. This was augmented by the notion that stress is well known to increase risk for relapse for those with this diagnosis (Hammen and Gitlin, 1997) and stress related to COVID-19 such as loneliness, uncertainty and being at a high risk for contracting COVID-19 due to comorbid health conditions related to BD (De Hert et al., 2011) may also increase deterioration of symptoms and the risk of relapse (Chatterjee et al., 2020).

Following the shift to online interventions, necessitated by the pandemic, new research has emerged into the delivery of remote group psychological interventions. However, this remains somewhat limited within the BD client group. Three recent studies discuss the feasibility and acceptability of online group interventions for BD (Gadelrab et al., 2022; Perich et al., 2024; Newton et al., 2025). A qualitative study of participants’ experiences of an online group wellbeing intervention for BD found an increased sense of connection, increased feelings of safety, reduced need for travel, and decreased barriers to engagement (Perich et al., 2023). A randomized controlled trial reported an improvement in perceived quality of life following a remote psychoeducational intervention for BD (Perra et al., 2024), providing evidence that online group interventions may be beneficial for this client group. Newton et al. (2025) found significant quantitative improvement in self-reported recovery following an online programme.

There is greater research of remote group therapy in other client groups, with a number of reviews published (Banbury et al., 2018; Carlbring et al., 2018). Gentry et al. (2019) found several aspects of face-to-face psychological services such as accuracy of assessments, therapeutic alliance and outcomes to be retained in telehealth, with some service users preferring online delivery (Batterham and Calear, 2017). These findings are echoed by Rafieifar et al. (2025) systematic review of randomized controlled trials, which found face-to-face and remote group interventions to be comparable in retention rate and reducing symptoms of presenting issues.

In exploring the differences between delivery methods, Lopez et al. (2020) found that the in-person group participants felt more connected to other group members than those participating online, resulting in a significant difference on the group cohesion scale. Despite this drawback, there was a considerably better attendance rate in the online group, which qualitative participant feedback suggests was due to the convenience of being able to meet online. This study emphasized the importance of considering whether the benefits of online delivery outweigh the costs. Greene et al. (2010) investigated alliance in an online anger management group compared to a face-to-face group. Results found that although the groups did not differ significantly on most process variables, those in the remote condition experienced a weaker alliance with the therapist than those in the face-to-face group. Moreover, individuals who had a stronger alliance with the therapist tended to have better anger reduction outcomes. However, it should be considered that those in the online group may have had preconceived ideas about the quality of online relationships and therefore underrated their connection with the therapist.

Another difference between online and in-person therapy delivery are technological issues surrounding online participation. Sansom-Daly et al. (2019) state that technological difficulties were common in an online peer support group for young adults recovering from cancer. Although the study reports the low burden and high benefit of the online group, it was only compared to a wait-list control. Therefore, it cannot be assumed that an in-person group would have had the same level of benefit without the technical problems causing hindrance. This study also highlights the question of equality for and diversity of those able to access an online group. For example, issues of digital poverty and internet illiteracy should be considered, thus meaning that much of the current literature may be unrepresentative of a wider sample. Hassija and Gray (2011) also focused on the concept of inequality regarding those living in rural areas who have limited access to mental health services. Positive outcomes in this study suggest that online therapy may increase equal access for those in rural areas.

Taken together, studies identify both potential benefits and challenges of online delivery for group programmes, which need further consideration for individuals with severe and enduring mental health conditions. This population is at a high risk of digital exclusion due to deficits in digital skills (Spanakis et al., 2024) possibly as a result of being at an increased risk of poverty, social isolation or psychotic symptoms such as paranoia (Bipolar Commission UK, 2022; Chakrabarti and Singh, 2022). There is an opportunity to improve digital literacy through online interventions, but it is important to understand service users’ experiences to improve accessibility to services and to reduce health inequalities. In previous studies, service users have not been in the expert position of having experienced a group intervention both face to face and online and able to compare these formats.

The current evaluation focuses on an NHS service (United Kingdom) specializing in psychological interventions for individuals with a diagnosis of bipolar disorder. The service provides a psychological programme called Mood on Track (MoT) which comprises group-based cognitive behavioral therapy (CBT) followed by individual relapse prevention sessions to create a ‘staying-well plan’. Prior to online delivery it consisted of 11 weeks of group CBT followed by 6–8 individual relapse prevention sessions. Jones et al. (2018) found that providing face to face Mood on Track in a mental health service increased access to psychological therapy by 54% for those with BD (when compared to the 6 years prior). For those in receipt of the intervention, personal recovery significantly improved from pre-to post-intervention. Secondary outcomes, such as quality of life, social functioning and mood and anxiety symptoms, also showed improvement post-therapy with smaller effect sizes.

The current study focusses on the group CBT element of the programme. This covers themes such as understanding low and high mood, relaxation, and relationships (see Table 1) and is in line with NICE recommended psychological intervention for this client group (National Institute for Health and Care Excellence, 2014).

**Table 1:** Session topics of the programme.

Session number	Topic
1	Service user perspective
2	Introduction to diagnosis and mood changes
3	Understanding high mood
4	Understanding low mood
5	The role of stress
6	Coping with high mood
7	Coping with low mood
8	Understanding your medication
9	Relationships and communication skills
10	Mindfulness and relaxation
11	Putting it all together and managing special situations

The pandemic offered the opportunity to trial and investigate online intervention. Prior to this service users were seen face to face within community bases and there was no infrastructure to support remote interventions. Circumstances during 2020 resulted in a group of participants who began the group face to face and then switched to online sessions, via the video-conferencing platform Zoom. Features of the intervention were adapted to the online format such as the weekly assessments, group activities and breaks thus it was imperative to gain in-depth insight into the clinical perspective of whether service users and staff felt their needs were met, their experience of this transition and the acceptability and feasibility of these adaptations.

As highlighted research to date lacks focus on the potential for delivering group interventions online for a high-risk BD population highlighting a need for evaluation. Given the dearth of such research, it is important to conduct qualitative investigations of feasibility and acceptability (Czajkowski et al., 2015) at this early stage of intervention development. The clinical questions explored within this preliminary exploration of feasibility and acceptability included:

Do professionals think it is possible to deliver therapy online?
Can professionals manage risk and keep individuals safe?
What are the challenges and benefits of online/face-to-face modes of delivery?
What are the barriers and facilitators related to each mode of delivery?
Do service users report therapeutic benefits across both platforms?
What are the considerations for engagement and delivery related to each mode of delivery?

These questions were reflected in the topics covered within interviews and the focus group.

The overarching aim of this evaluation was to provide qualitative insight into service users’ and staff members’ experiences of the feasibility and acceptability of online as compared and contrasted to face-to-face CBT intervention for Bipolar Disorder. These insights may contribute to how similar interventions for those with a diagnosis of bipolar disorder may be delivered in the future.

## 2 Materials and methods

### 2.1 Design

A qualitative method was used to provide an in-depth, contextualized understanding of individual perceptions and experiences of two contrasting group delivery formats, namely face to face and remote. Semi-structured interviews and focus groups were used. Service evaluation approval was obtained through the local NHS trust.

The research team consisted of senior clinical psychologists, undergraduate and assistant psychologists, psychotherapists and researchers. Those undertaking the analysis worked with the Bipolar Service and therefore may have had some positive bias. To reduce bias the interviews were undertaken by an experienced qualitative researcher and psychotherapist who was not working within the NHS and analysis was overseen by the Director of Research who did not work in the service. The research team all had psychological backgrounds and training.

### 2.2 Participants and recruitment

All participants were under the care of Community Mental Health Teams (CMHT) in secondary care mental health service within the NHS in England. They were referred by their consultant psychiatrist or care coordinator to the specialist psychological therapies service for Bipolar Disorder to participate in the Mood on Track programme. The criteria for the programme are that service users have a diagnosis of Bipolar Disorder (BD) or experience high and low mood severe enough to disrupt day to day functioning and following a structured clinical conversation with a therapist have opted to attend Mood on Track. The sample for this study was opportunistic, they were a subsample taken from ten people attending the Mood on Track programme which began in February 2020.

This programme was initially delivered face-to-face in February 2020 and was temporarily halted in March 2020 when the first national lockdown was implemented in the UK. At this time, six of eleven group CBT sessions had been completed. All ten group members were given the opportunity to complete the intervention programme in an online group or via individual telephone sessions. Group sessions resumed online in June 2020 once the programme had been adapted for online delivery and video technology was in place. Eight of the original ten group members opted to complete the group online. One of these members left the group due to ill physical health, leaving seven participants who were invited to participate in the qualitative evaluation. Six of the seven individuals consented to being involved in the evaluation. All participants had a diagnosis of Bipolar Disorder. The three group facilitators were also given the opportunity to attend a focus group about their experiences of facilitating online and face-to-face group therapy. All agreed to participate. See Table 2 for participant information.

**Table 2:** Demographic data of service users and staff.

Demographic characteristic	Service user sample (*n* = 6)	Staff sample (*n* = 3)
Age, years: M (SD), Range	40 (9.67), 33–58	43.7 (13.6) 28–52
Gender		
Female	33.3%	100%
Male	66.6%	
Ethnicity		
Mixed	16.6%	
Black Caribbean	16.6%	
White	66.6%	100%

### 2.3 Materials

Researchers developed two semi-structured interview schedules, one for the service user interviews and one for the staff focus group. Both schedules covered attending/facilitating face-to-face sessions, attending/facilitating online sessions, and individuals’ preference for online versus face-to-face. The staff focus group interview schedule explored facilitators the experience of moving online and compared both forms of delivery. See Table 3 for more information.

**Table 3:** Interview guide.

Topics	Service user interview	Staff focus group
Before the group	Expectations and hopes	Aims Anticipated challenges and difficulties
Face to face group	Helpful/unhelpful aspects Challenges or areas for improvement	Helpful/unhelpful aspects Challenges or areas for improvement
Lockdown	Experience of pausing the group Impact on symptoms	Experience of pausing the group Contact with group members Development of the course moving online Obstacles/facilitators of moving the group online
Online group	Overall experience Helpful/unhelpful aspects Challenges or areas for improvement	Overall experience Helpful/unhelpful aspects Challenges or areas for improvement
Comparison	Benefits and drawbacks of the two group formats	Benefits and drawbacks of the two group formats

Researchers from outside the service, alongside the psychologist within the service developed the interview schedule. Researchers included an experienced qualitative researcher and an expert in qualitative methodology. The interview schedule was reviewed by the manager of the service, who has expertise in both qualitative methodology and psychological interventions for bipolar disorder.

### 2.4 Procedure

Following completion of group CBT all group members were given an information sheet and the opportunity to participate in an interview. 6 participants were interviewed about their experience of attending online and face-to-face group completion of the group and therapy the three group facilitators were also given the opportunity to attend a focus group about their experiences of facilitating online and face-to-face group therapy.

The interviews and the focus group were conducted remotely by video call once the group was completed. Both the focus group and individual interviews were approximately 45 minutes in length. Both were conducted by a researcher outside the service. External researchers were selected to reduce the potential for researcher bias and reduce the likelihood of demand characteristics from the service users and facilitators being interviewed.

Each interview/focus group was recorded and verbal consent to record was taken at the beginning. Consent was also documented on each service users’ patient record. Participants were informed that they could withdraw at any point and that this would not affect their care and that all data collected would remain confidential and anonymous.

### 2.5 Data analysis

The interviews and focus group were transcribed verbatim and anonymized. Pseudonyms were given. The two data sets (the interviews and the focus group) were analyzed separately by different members of the research team. Both data sets were analyzed using thematic analyses, following closely the guidelines developed by Braun and Clarke (2006). Researchers familiarized themselves with the data and developed initial codes. A semantic approach was taken, extracting the surface meaning of the dataset rather than examining the underlying assumptions. When searching for and identifying themes, an inductive approach to thematic analysis was adopted. Themes had to be relevant to the research question. The datasets were reviewed twice to ensure full consideration was given.

In order to ensure the plausibility and credibility of the analysis the coded transcripts were then read by an additional member of the research team to ensure consensus. This method of triangulation was used to increase inter-rater reliability and reduce bias. Furthermore, the themes from both analyses were reviewed and refined in discussion with the whole research team.

## 3 Results

The findings from service user interviews and the staff focus group are presented separately, with accompanying data extracts. The thematic analysis conducted on the service user interviews generated three superordinate themes with a number of subthemes. Analysis of the staff focus group also resulted in three superordinate themes, each with underlying subthemes. Themes across both analyses are compared in Table 4.

**Table 4:** Table comparing topics discussed in both service user interviews, and the staff focus group.

Topic	Service user interviews	Staff focus group
Relationships with other group members	Regardless of the delivery type, service user reflected on how meeting others helped them to normalize their condition. Being face-to-face was perceived as an important factor to facilitate social bonding. The transition to remote delivery was perceived to negatively impact how relationships progressed, with breaks being seen as rigid and sessions lacking informal interaction.	Staff discussed how remote delivery created opportunities for service users to talk to different group members through breakout rooms. Staff highlighted the benefits of face-to-face delivery for group bonding, including being in close proximity with each other and increased informal bonding opportunities.
Relationship between group members and staff	No comment from service users	Staff described struggling with a loss of therapeutic connection online, difficulty reading non-verbal cues and difficulty retaining service user information. They did highlight one bonding opportunity being the collaborative experience of learning about virtual delivery of the intervention together.
Accessibility of the course	Service users explained how the online format was more accessible for them in relation to travel difficulties, and mental health or physical health conditions.	Staff discussed how remote delivery was more accessible for individuals struggling with transport or mental health difficulties. Staff highlighted the need to add an extra session for individuals to get to grips with the video platform. Staff did share concerns about digital poverty.
Concerns for safety	Service users highlighted difficulties finding a confidential and private space when the course transitioned online.	Staff shared concerns about how risk would be appropriately assessed online. They also shared concerns regarding ensuring confidentiality within the virtual setting and the importance of choosing an appropriate video platform.
Difficulties adapting to online delivery	Service users discussed the importance of some activities being completed face-to-face and highlighted difficulties with them being delivered remotely.	Staff highlighted issues with the transition from face-to-face to online including having to tailor the course content, increased client check-ins and adding more sessions. These difficulties all added to staff workload.
Impact of the intervention on recovery	Without referring to the delivery type, service users described how the course helped them to think differently and helped them to better manage their symptoms.	No comment from staff
Ability to engage	Service users discussed how the symptoms of bipolar disorder affected their concentration and ability to engage. Some individuals reported preferring online delivery to aid concentration whilst others said it was easier face-to-face.	No comment from staff
The future of the intervention	No comment from service users	All staff members expressed the desire to continue with a hybrid model of delivery, highlighting advantages of both delivery methods.

### 3.1 Service user interviews

Three main themes were derived from interviews with service users. Each had several subthemes.

Theme 1: Challenges with engagement and adaptation, with subthemes (a) ability to engage and participate and (b) lack of flexibility in online delivery.

Theme 2: Personal impact of the intervention, with subthemes (a) social bonding and (b) changes in thinking and symptom management.

Theme 3: Barriers to accessibility, with subthemes (a) difficulty of travel and (b) finding privacy and ensuring confidentiality.

Each theme and the subthemes within it are described below. Pseudonyms have been used to preserve anonymity.

#### 3.1.1 Theme 1: Challenges with engagement and adaptation

The first theme that emerged from the service user interviews focused on the challenges that arose when attending the group (both face-to-face and remotely). Two subthemes evolved: (a) ability to engage and participate, and (b) lack of flexibility in online delivery.

##### 3.1.1.1 Ability to engage and participate

Irrespective of how the course was delivered, service users reported how symptoms of bipolar disorder affected their concentration and ability to participate in the group sessions. Mixed opinions arose regarding the preferred delivery method for aiding concentration. One individual explained that face-to-face delivery was *“probably easier to focus and make sure that you had dedicated time (Andria, 481–482),”* however another individual noted distractions during face-to-face delivery.

*“The room was small, it would get overheated in winter, the window would have to be opened and of course as soon as you open the window you would get construction noise.” (George, page 7)*

One service user found that online delivery helped to aid concentration, *“when it’s on the iPad and here you go, this is what we are looking at, I mean that draws focus very well (Daniel, 360–362).”* However, the chat function on Zoom was described as *“a bit of a distraction (Andria, 417–418).”*

One individual discussed their preference for online delivery when feeling mentally unwell:

*“I probably found it easier when feeling a little bit low to still get on an online session rather than get out of the house and get all the way to a face to face.” (Andria, page 19)*

##### 3.1.1.2 Lack of flexibility in online delivery

Service users commented on how certain activities were best delivered face-to-face and how the service struggled to adapt these to an online format.

*“I am thinking of things like the card exercises […] we would kind of maybe do things in small groups for a little while for a few minutes and I guess that kind of thing was good being in the same room together (page 6) …I felt some material (online) was rushed through and it was not being able to do some of the exercises.” (page 16). (Stacey)*

It was also mentioned that when the group intervention was delivered online the sessions felt too short.

*“For some reason the two hours felt like they went really quickly…they felt like they just flew past, and it felt like the sessions were too short.” (Sam, page 14)*

#### 3.1.2 Theme 2: Personal impact of the intervention

Service users reflected on several elements they felt that they benefitted from and whether they were unique to one delivery method or consistent across both. These reflections were characterized into the following subthemes: (a) social bonding, and (b) changes in thinking and symptom management.

##### 3.1.2.1 Social bonding

Service users shared that the group format allowed them to connect with other people who had a diagnosis of BD and shared similar experiences. Without referring to the format in which the group intervention was delivered, service users reflected on how meeting others helped them normalize their condition and feel less alone.

*“Being in a group of you know eight – ten people who all have similar experiences you know it does help with that not feeling so alone.” (Daniel, 68-70)*

*“Some of them were just, ‘I get that, and I get that’ type of conversation which makes you feel, I say normal.” (Sam, 393-39)*

Being physically in the same room was perceived to be an important factor to facilitate social bonding, with one service user commenting on how being in a small room together contributed to how closely they bonded.

*“I think there is something to being in a room with other people, I think there’s sympathy and radio waves and brain shared consciousness and all that kind of stuff.” (Sam, 581-584).*

Many service users believed that meeting face-to-face for the first half of the group helped them to build rapport.

*“When that transition happened, I was pleased to see that the group dynamic remained the same as well (91-93) … I think that’s why we all found that transition easier once the course had changed because we had all met one another in person and we had already formed a group dynamic.” (392-394). (George)*

*“You might feel like you know each other better after the one in person and you might feel like ‘do you want to go for a coffee or something’ after you have finished.” (Stacey, 469-472)*

The transition to remote delivery was perceived to have a negative impact on how social relationships progressed. Refreshment breaks over Zoom were reported to be rigid and lack the interaction and camaraderie shared in face-to-face refreshment breaks.

*“…it had to be a little bit more rigid in how we all interacted because we couldn’t have it [the refreshment break] as a group […] I just don’t think it works as well, literally people would disappear.” (Sam, 374-380)*

##### 3.1.2.2 Changes in thinking and symptom management

Without referencing the delivery method of the group intervention, service users expressed the thought-provoking nature of each session, highlighting its ability help them *“think in different ways”* (Sam, 148–149). One service user mentioned how they would be in contemplation hours after their session and continued to think about what they had learnt.

*“Meeting up for a couple of hours to go ‘this is why your plans not working or this is why you messed up’ would actually leave me with a couple of hours of contemplation afterwards … you know I wouldn’t walk out of sessions and then go ‘right that’s it’ for seven days and not think about it. Tuesdays were a day where I end up sitting and thinking about it.” (Daniel, 159-165)*

Irrespective of delivery method, service users mentioned how the group intervention helped them understand more about bipolar disorder, their treatment and have more control over their symptoms.

*“To just start from a point of positivity of symptom management and not to just see this as something that happened to me and now, I’ve kind of got to deal with it, it made it a lot more positive and a reflective process.” (Andria, 528-532)*

*“I learnt to identify lows really quickly … coping with depression I found there was loads of strategies and things I could do to try and help.” (Stacey, 203-206)*

*“We had a visit from somebody who did the medication, and he was great, we were able to ask questions and stuff and find out about what we were taking, and I found it very useful.” (Isaac, 241-243)*

#### 3.1.3 Theme 3: Barriers to accessibility

Service users commented on two key barriers which impacted the accessibility of the intervention: (a) difficulty of travel, and (b) finding privacy and ensuring confidentiality.

##### 3.1.3.1 Difficulty of travel

Some service users expressed that the online format was more accessible to them (due to having visual impairment or severe anxiety) and discussed some of the challenges with attending face-to-face.

*“…you know usually it takes a little bit more time to work out how to get to a particular location if I am not terribly familiar with it, especially if you experience sight loss, you know that can be tricky.” (George, 147-149)*

*“There were times when I felt like it was hard work, really hard for me to go and I used to get anxiety about it…and the night before I wouldn’t sleep very well (149-154)… Bus wise I would of found it difficult, there wasn’t a direct route, I would have had to kind of give myself at least an hour and a bit to get there.” (169-171). (Isaac)*

One service user also recognized how driving is a common barrier faced by those with BD due to strict Driving and Vehicle Licensing Agency regulations and noted how this may impact upon ability to travel, *“obviously people with Bipolar like myself at times cannot drive” (Sam, 264–265).*

##### 3.1.3.2 Finding privacy and ensuring confidentiality

Some service users shared difficulty in finding a private and confidential space when the course transitioned to remote delivery. Having an agreed safe word with the group, which meant that someone was nearby whilst the service user was accessing the group online and everyone in the group remained quiet when the word was used was noted as reassuring for service users.

*“It was a bit difficult at first at my sister’s, but we managed it, found a nice spare room that I could use and we had words that if anyone came in the room we had like a kind of safe word we could use for confidentiality”. (Isaac, 224-227)*

*“I was skeptical that I wouldn’t be able to make the same time and space for it, you know find somewhere separate to go that didn’t have, what was sort of private enough” (Andria, 332-334)*

### 3.2 Staff focus groups

Three main themes were derived from the focus group with facilitators. Each had a number of subthemes:

Theme 1: Bonding, with subthemes (i) group relationships, (ii) lack of informal bonding and (iii) therapeutic relationships.

Theme 2: Inclusivity, with subthemes (i) service user ability and (ii) digital poverty.

Theme 3: Practicalities of delivering online, with subthemes (i) staff workload and wellbeing, (ii) safety of service users, and (iii) flexibilities of the intervention.

Each theme and the subthemes within it are described below. Pseudonyms have been used.

#### 3.2.1 Theme 1: Bonding

This theme focused on how individuals in the group, both staff and service users, were able to make connections with each other, and how this differed between the face-to-face and online setting. This theme is broken down further into subthemes: (i) group relationships, (ii) lack of informal bonding, and (iii) therapeutic relationships.

##### 3.2.1.1 Group relationships

Staff members talked about how bonding between service users differed depending on type of delivery. Online, service users were put into smaller breakout rooms, and this was decided by the group facilitators. However, when the group was face-to-face individuals would sit in the same seat and talk to the same individuals each week. Staff discussed how using breakout rooms created opportunities for service users to talk to different members of the group and build new relationships.

*“On zoom we put them into breakout rooms … we keep mixing it up and so actually that would never have happened with face to face that they are getting to chat to other people in the group a bit more.” (Becky, 541-546)*

Conversely, staff also highlighted the benefits of the face-to-face setting and the impact that this had on group bonding. The room used for face-to-face groups was small in size which meant service users were *“almost sitting on top of each other” (Laura, 134–135).* This close proximity was regarded as potentially influencing the formation of positive group relationships, *“I am wondering whether that had any impact on the group bonding because they had to sit so close to each other” (Becky 142–144).*

##### 3.2.1.2 Lack of informal bonding

Staff members highlighted that the online group afforded limited opportunities for informal conversations. Staff stated that in the face-to-face group breaks were a good opportunity for small talk, compared to the online breaks where everyone would turn their cameras and microphones off. Staff emphasized the importance of physically being together in a room. They explained that when the group was delivered face-to-face, service users often arrived early or left late, which also provided a chance for informal discussions. Staff members considered this to be important in strengthening relationships between group members as well as between staff and service users. Staff members viewed such situations as impossible to duplicate in the virtual setting.

*“They come to the group and then they go, and I just don’t think there’s that hanging around or being sort of almost forced into being together because they are physically here.” (Jane, 783-786)*

*“Even though we open up the room at ten to [online], not many of them come in early to have a chat.” (Becky, 773-774)*

One staff member questioned how service users connect as a group online and predicted that *“perhaps when the online group ends their online interaction will end.” (Jane 759–760).*

##### 3.2.1.3 Therapeutic relationships

Staff members talked about how the relationship between themselves and the service users was affected by the nature of the online group. They disclosed struggling with a loss of therapeutic connection.

*“You know face to face I can talk through ten slides and not feel that I have to stop and chat but on zoom I say you get to four or five and I want to stop the PowerPoint and just see people and chat and I do feel you miss a bit of that connection.” (Becky, 179-483)*

Staff members also highlighted how online delivery made it difficult to pick up on non-verbal cues, causing staff to feel a further loss of connection, *“you do not get the same sense in terms of body language that you do with in the room experience… when we reflect on what happened in the session, because normally we would be able to think about the position people were sitting… but remotely you do not get that.” (Laura, 484–493).*

Staff members also expressed difficulties retaining information about group members when delivering the group online, due to a loss of visual cues.

*“What are we, in week five and I still can’t remember who is in what group and I normally would know but at the moment I have to really stop and think oh who’s that again?”(Becky, 498-501)*

One positive of online delivery highlighted by staff, referred to clinicians’ uncertainty using Zoom which provided an opportunity for collaborative learning with the service users. Staff noted how this helped with the dynamics of the group and assisted with building therapeutic relationships with a sense that they were all ‘in it together’.

*“Something quite nice in that everyone … is like helping hands … it also helped perhaps in some ways build relationships further because people were just, we are all muddling in it together.” (Jane, 563-569)*

#### 3.2.2 Theme 2: Inclusivity

Staff discussed access to the service and identified challenges to be addressed to ensure the course was accessible to all. This theme is split into 2 subthemes, (i) service user ability, and (ii) digital poverty.

##### 3.2.2.1 Service user ability

Staff members discussed how the course accommodated service users with differing abilities, including digital ability and health difficulties. They described making practical accommodations during the face-to-face sessions for an individual with a visual impairment, including having to *“describe things to [them] and special considerations for filling out forms” (Becky, 123–124).* Staff went on to explain that when the group was face-to-face, *“group members made [this service user] a cup of tea… that kind of helped with the kind of sense of warmth and connection.” (Laura 126–128).*

Staff focused on one advantage of online delivery being that it could be more accessible to service users *“who do not like leaving the house or have difficulty with transport” (Becky, 611–613).* Staff stated that face-to-face sessions may *“feel too much” (Laura, 899)* for individuals with high anxiety, explaining that they may be more inclined to *“give it a go meeting with the remote group” (Laura, 987–898).* The online chat function and virtual break out rooms were described to benefit one service user who was very anxious.

*“She is using the chat function, and we are reading out her comments and she is still really actively engaging … in a face to face setting she would sit and get a bit lost.” (Jane, 374–878)*

When delivering the group online, staff members highlighted participant’s differing digital abilities and how this impacted the running of the group. This resulted in adding an additional session to overcome challenges.

*“We gave them an extra week to set it up and check everyone because some people had already been using it [zoom] with work, some people had used it prior to all this but there were some people that hadn’t.” (Jane, 402-405)*

##### 3.2.2.2 Digital poverty

Staff members shared concerns regarding the accessibility of the course when delivered online, especially in relation to digital poverty.

*“We are excluding … not a lot of people - but we are excluding people because they haven’t got access.” (Becky, 589-591)*

Staff members also identified drawbacks of accessing the group without appropriate equipment.

*“Meeting on a phone is not the same as having it on an iPad or laptop where they have got a good screen like we have (Laura, 594-595) … Where they can just sit it in front of them instead of moving around and holding it there because that’s what you do with a phone.” (Becky, 596-298)*

Staff thought that service users would *“appreciate the fact that we are trying to keep things going”* (Laura, 224) during the pandemic however ideally being able to provide the option of both a face-to-face and online group was considered by staff members as being core to the inclusivity of the service in future. Allowing the format to be best suited to service users’ needs and access to equipment would allow more individuals to access the course.

#### 3.2.3 Theme 3: practicalities of delivering online

This theme discussed the viability and feasibility of delivering the programme online. It is split into three subthemes: (i) staff workload and wellbeing, (ii) safety of service users, and (iii) flexibilities of the intervention.

##### 3.2.3.1 Staff workload and wellbeing

Transitioning from face-to-face to online had multiple challenges for what was described as *“very small staff group” (Laura, 634).* Staff reported having to adjust the therapeutic course material and delivery in order for it be offered online, inevitably increasing their workload.

*“Stressful … it all happened very fast; I think I recall we had a sort of an emergency team meeting about what we were going to do.” (Jane, 170-172)*

They also expressed concerns about the increase in the amount of time required when delivering online, which was reported to increase pressure on clinicians. Staff members stated that they had to check in more with clients online which *“slow[ed] the pace of the group” (Jane, 648–649)*.

*“It was 11 weeks, we have now made the groups 13 weeks … that’s an extra six weeks a year that we are doing groups for and if we extend it any more than that, that’s going to eat into individual time.” (Becky, 676-685)*

Staff reported feeling concerned about how the service users would cope during the transition to working online, describing this as a *“horrible feeling” (Jane, 179).* They expressed feeling motivated to keep going but felt stuck in *“a balancing act between what we perhaps would want to do and what we can feasibly do” (Jane, 688–689).*

##### 3.2.3.2 Safety of service users

One of the main considerations for online delivery was the safety of service users. Staff shared worries about how the outcome measures (which were used to assess risk and wellbeing) would be delivered during the online sessions.

*“Outcome measures that we do on a weekly basis, a lot of them like to assess risk like the PHQ-9 how are we going to do that, how are we going to ensure people are safe?” (Jane, 314-316)*

Another prominent safety concern was how to ensure confidentiality within the virtual setting. As service users would be joining from within their own home, staff members had to consider extra measures that would need to be taken.

*“In terms of people being able to guard their space so in terms of confidentiality so we had to think a lot about… the planning and the meetings so that the sessions were as secure as possible really so I think we did put a lot of time and thinking into that” (Laura, 388-392)*

There were also challenges that had to be resolved in finding an appropriate platform. Staff discussed difficulties with Microsoft Teams and the need to source a license for using Zoom.

*“You could have something like six people on the screen which would mean there would be no possible way for us to conduct a group because our group at that time I think had seven or eight people.” (Jane, 268–271).*

##### 3.2.3.3 Flexibility of the intervention

Throughout the focus group, staff members discussed how the course may look in the future. All staff members expressed the desire to continue with a hybrid model of delivery, explaining that there were advantages to both face-to-face and online delivery.

*“I do really like being able to do zoom so it would be nice to … be able to offer the whole variety and be driven by service users wishes or needs.” (Becky, 861-864)*

Staff shared that working online provided more gaps in the working day which helped to relieve pressure. They also highlighted the benefit of reduced travel time. One staff member reported that online working makes them “*more productive as a service” (Laura, 848).* Staff members stated that the flexibility of online delivery allowed them to *“mix up the groups according to clinical need… we do not have to worry about their geographical constraints” (Becky, 622–626)* where previously groups ran in specific localities which could lead to inequitable waits in more populus areas.

## 4 Discussion

As far as the authors are aware, this is one of the first studies to explore the experiences of service users and staff who have participated in both online and in-person group CBT intervention for bipolar disorder having experienced both modes of delivery *within* the same intervention (because they switched from one to the other during the pandemic). Whilst this resulted in a small sample it constitutes one with a unique perspective and insight. The study highlights advantages of delivering the programme online, including increased accessibility for individuals with mental and physical difficulties, as well as some considerations for remote delivery, including confidentiality, risk assessment and challenges with the therapeutic relationship. Each of the study findings are discussed in further detail below.

Both service users and staff discussed the importance of being in the same room and within close physical proximity to each other for ease of building relationships and aiding social bonding. Both groups also discussed the rigidity and lack of informal bonding opportunities in online groups (especially before and after the group, and during break time). These findings support previous research by Lopez et al. (2020) which found that individuals who received face-to-face group therapy felt more connected to the other group members compared with those participating online. These findings raise some challenges about the feasibility of online delivery for building effective relationships.

Although service users in the present study reported increased difficulty with building relationships online, they still highlighted the power of connecting with similar individuals and therefore still appear to be benefitting from this bonding and normalizing experience online. This is a particularly important finding for those who cannot attend a group in person, especially given the high levels of social isolation in this group. It is also beneficial when it is not practical for a service to offer this, for example in rural locations. Perich et al. (2023) reported that participants living in rural locations benefitted from the opportunity of connecting with others with a BD diagnosis through an online group. This also supports the findings from a previous study conducted by Gerrits et al. (2007) which found that group members participating in an online chat group intervention for depressive symptoms developed a strong bond and wanted to share contact details following the study. However, it is important to highlight that individuals in the present study had already formed face-to-face relationships before transitioning online; different results may be reported by individuals who complete the course solely online.

In the present study, staff highlighted a loss of therapeutic connection online, due to feeling as though they were talking to a screen, struggling to retain service user information, and missing non-verbal communication. Previous studies exploring the effectiveness of online interventions have largely ignored the impact on therapeutic relationships (Sucala et al., 2012). Morland et al. (2011) reported that individuals who participated in an online anger management group felt a weaker alliance with their therapist compared to those participating in a face-to-face group and some research has demonstrated challenges with emotional connection online (Békés et al., 2021; Rees and Stone, 2005) which is in line with what therapists in the present study suggest. Moreland’s study also found that individuals who completed the group face-to-face also went on to have larger anger reduction outcomes compared to the online group. The recovery outcomes in the current study were not investigated but this will be important to consider in future research. Future research should also include a measure of therapeutic alliance as this may influence outcomes (Alldredge et al., 2021).

In contrast, other studies have suggested that a positive therapeutic alliance can be formed in online interventions and can bring advantages that face-to-face cannot, for example service users may find the online environment more comfortable and less threatening (Kocsis and Yellowlees, 2018; Reynolds et al., 2013). Certainly, in the current study it was highlighted that it was easier for some service users to attend from home, such as those with disabilities or anxiety. Aafjes-van Doorn et al. (2021) reported that although therapists felt less connected to their patients online compared to in-person, they still rated this as a good therapeutic relationship. One bonding opportunity that staff did emphasize in the present study was the collaborative process of learning to use a video platform alongside the service users providing a sense of ‘all being in it together’.

In the present study, service users and staff also discussed increased accessibility of the online group for individuals feeling unwell (e.g., severe anxiety, visual impairment, mood changes) as well as the elimination of transportation difficulties and associated costs. These findings highlight benefits of delivery of online interventions. Previous research suggests that meeting online may be more convenient and accessible for individuals, increase attendance and increase access to a variety of therapists (Cipolletta et al., 2018; Lopez et al., 2020; Stoll et al., 2020; Xue et al., 2020). Staff members in the present study did however highlight that online delivery may exclude individuals, due to lack of equipment, technical difficulties or digital ability. Previous studies have explored the equality and diversity of individuals able to access an online group (e.g., digital poverty, internet illiteracy) and questioned whether the current literature is representative of those who cannot engage in online therapy (Hassija and Gray, 2011; Sansom-Daly et al., 2019). Ethnically minoritized groups experience increased barriers to accessing services in the UK (Memon et al., 2016) and may be less likely to be offered a range of evidence-based interventions (Das-Munshi et al., 2018) and further research is needed in how this might be compounded by the shift to online service provision.

Although not an issue reported by this particular group, technological difficulties have been reported as common in research studies and have been found to affect the flow of the session (Cipolletta et al., 2018; Sansom-Daly et al., 2019). Therapists did highlight the importance of considering and acquiring access to the most suitable video platform for online delivery which may have minimized such issues.

Both service users and staff in the present study highlighted the difficulty of finding a private space and concerns for confidentiality. This has previously been raised as a challenge (Békés et al., 2021; Lustgarten et al., 2020; Stoll et al., 2020) in research resulting in service users feeling uncomfortable talking when others are in their house (Cipolletta et al., 2018). A safe word was used in the current study and this appeared to reduce the risks of breaching confidentiality and helped service users to feel safe.

The final theme outlined by both service users and staff focused on the transition to online delivery. Staff talked about their increased workload, setting up the course and making adaptations for online delivery and service users talked about the difficulty in translating some of the exercises into an online format. The use of break-out groups and mixing service users up in new combinations was mentioned as one positive of this transition.

Previous research exploring the transition to online therapy is limited, however a review exploring ethical issues in online psychotherapy reported a common theme across studies being that online delivery offers greater flexibility for therapists in terms of location and time and creative online material and modalities for service users (Stoll et al., 2020). Connolly et al. (2020) reported that concerns about online therapy tend to decrease following experience of using it and this may be the case if further research was conducted with the participants from this study.

A number of topics were further covered in the service user interviews but not the staff focus group. Service users discussed how irrespective of delivery type, the course helped them to think differently about their condition and recovery and helped with symptom management, supporting previous research (Chiang et al., 2017; NICE, 2014; Watson and Dodd, 2017). This provides evidence suggesting that the intervention may be effective both online or face-to-face for people with a diagnosis of Bipolar Disorder. Quantitative evaluation comparing outcomes of face-to-face and online intervention would be beneficial to further investigate this. Service users did report that their symptoms of bipolar disorder, such as difficulty with concentration, affected their level of concentration in the group, with some individuals preferring face-to-face delivery to minimize distractions and others preferring online. Therapist and service user distraction during sessions (such as self-view on video technology, lack of distinction between therapy time and home time, zoom fatigue and notifications on their device) have been proposed as challenges of online psychotherapy (Békés et al., 2021; Brainard and Watson, 2020). This is further evidence for offering choice of online or face to face intervention in future.

Staff members discussed topics that were not present in the service user interviews. They expressed concern for gauging and establishing risk online, specifically how outcome measures would be adapted for online use and how non-verbal cues could be missed. This is particularly important when working with those with this condition. This links with the previous theme and literature discussing difficulties with feeling connected to service users (Békés et al., 2021; Rees and Stone, 2005). Previous research has also highlighted challenges in reading emotions of service users online (Békés et al., 2021) which adds another barrier to effectively accessing mental health and risk, especially in a group setting. Direct messaging between service user and therapist was highlighted as a helpful tool for managing some situations in groups. The service has since worked to develop processes to address some of the concerns highlighted such as developing robust online risk assessment procedure using online outcome measures returned via MS Forms.

Finally, staff expressed a desire for a hybrid model of face-to-face and online delivery of the intervention moving forward, giving service users the choice in their care and widening inclusivity. This approach is increasingly being explored across mental health services and in research (Dunn and Wilson, 2021; Wentzel et al., 2016) and is advocated by the department of health in the UK.

### 4.1 Clinical implications

It is important to consider the feasibility of running online vs. face-to-face group interventions and consider potential implications for clinical practice. In this paper, increased difficulty in building relationships with other service users has been emphasized. Therefore, it is important for clinicians and services to consider how to increase opportunities online for informal and social bonding when taking therapies online. In this study, clinicians tried to overcome this difficulty by introducing more breakout rooms into the sessions to allow service users to discuss both session-related content and allow them opportunities for informal conversations. Clinicians also described frequently changing which service users were put together in break-out rooms to increase communication with all group members and adding additional sessions online to allow for more opportunities for discussion and checking understanding when presenting material. One important finding to highlight is that although relationships were reported as harder to build online, they were still able to flourish whilst online with service users reporting the positive effects and power of meeting with other individuals struggling with bipolar disorder. This finding supports the feasibility of online delivery for replicating the normalization effect of being in a group with individuals who have shared similar experiences. However, it is important to remember that service users in this study had already met face to face and attended the group together in person for six weeks, this is likely to have assisted with their bonding experiences. Future research may need to control for this to develop a clearer understanding of how relationships develop when remote contact is the only format experienced.

Similarly, staff highlighted difficulties building a therapeutic alliance with service users online. The therapeutic relationship between service users and clinicians has been found to be an important predictor of psychological treatment success (Castonguay et al., 2006; Morland et al., 2011) as well as predicting positive attitudes toward the efficacy of online therapy (Békés et al., 2021). Additionally, staff found it more difficult online to assess client risk. Therefore, it is important that services and professionals consider ways to do this for example collecting outcome measures via e mail and increasing one-to-one calls with service users pre and post group if risk concerns or changes in mental state are indicated, whether in the group setting itself or highlighted by outcome measures. These are changes which have been incorporated by this service since conducting this research study.

Online delivery was reported to increase accessibility for individuals with mental or physical difficulties as well as those struggling with travelling. This provides evidence for the feasibility of online delivery. However, digital poverty, technical difficulties and digital ability were highlighted as possible challenges with delivering therapy online. These factors must be considered carefully within mental health services and are thought to particularly impact care in low- and middle-income countries, however a review by Naslund et al. (2017) found that the use of online interventions in such countries may be feasible and acceptable and can helpfully support access to services. The possibility of introducing a hybrid model of care into services and offer choice may mitigate some of these factors and offer increased access to interventions to some groups.

Confidentiality was an important issue raised in this study which must be considered within clinical settings. In this study, the introduction of a safe word, when confidentiality had been broken in anyone’s setting, was used to help regulate confidentiality.

Clinical services must consider the time and flexibility of their service and staff. Whilst online delivery is thought to free up clinician time (Stoll et al., 2020), the service must spend time carefully planning how their intervention will be adapted to online delivery and consider what can and cannot be done in the online format. Since having conducted this service evaluation, the service has made a number of improvements to its online delivery of this group intervention programme and updated their intervention manual and resources guided by this feedback. One positive clinical implication which made the service more equitable was the ability to move to one online waiting list rather than having different wait lists in different geographical locations. One can imagine this being of significant benefit in more rural locations or in services where there are limited psychologically trained staff where staff members can see groups of service users online who previously would not have been able to access a service at all.

Finally, service users reported that overall course helped them with recovery and symptom management, irrespective of delivery method. This is a very promising finding which supports the intervention’s aims and the online delivery of the course.

### 4.2 Further research and limitations

Whilst this research study incorporated the views of both clinical staff and service users, it is important to acknowledge the small sample size. Only three staff members, who were all white British women were interviewed; therefore, results should be interpreted with caution. Although the service user sample was larger and more diverse, it still captures the views of a small proportion of individuals diagnosed with bipolar disorder. Future research should be conducted with a larger sample of individuals diagnosed with bipolar disorder to shed further light on the effectiveness and feasibility of online therapy for this client group. In addition this study was conducted within a specialist service setting in one area of the NHS in the UK and service delivery models in other countries may present additional barriers or facilitators to the feasibility and acceptability of online group interventions. In terms of information power (Malterud et al., 2016) future research with more diverse samples should be undertaken to add to our knowledge about other health services and delivery within these settings.

Additionally, this research study focused specifically on a CBT group intervention developed for bipolar disorder. The results therefore should be considered with caution across other psychological interventions or other mental health conditions.

Finally, this was an opportunistic evaluation presented at an uncertain time in mental health services. Whilst it provided us with a unique opportunity of exploring the change in group delivery format from the same service users and staff’s perspectives, it could be argued it is limited in its scope given the unique set of circumstances at the time of the evaluation. Although the findings from this qualitative study are rich and informative, it is also important to consider quantitative outcomes. Future mixed-method research should explore the effectiveness of online group interventions for bipolar disorder which includes quantitative outcomes measures, for example exploring changes in mental health, recovery and quality of life. It would also be beneficial to compare how outcomes (such as mental health, recovery) differ between delivery types.

## 5 Conclusion

In line with the study aims, staff and services user’s experiences of a remote and face-to-face group CBT intervention for bipolar disorder were explored, highlighting considerations for services moving forward. This study provides support for the feasibility of delivering this group intervention online in regard to recovery and symptom management, accessibility and normalization of bipolar disorder. However, the challenges and considerations of delivering this intervention online cannot be ignored, including time spent planning for online therapy, confidentiality, digital poverty, the therapeutic relationship and assessing client risk. Moving forward, services could consider the potential for offering both face-to-face and online delivery options, giving service users a choice in their care and widening inclusivity. Further research with individuals with a diagnosis of bipolar disorder or mood fluctuations is needed to help shape the future of delivering group interventions for this client group post pandemic.

## Data Availability

The raw data supporting the conclusions of this article will be made available by the authors, without undue reservation.

## References

[ref1] Aafjes-van Doorn K. Békés V. Prout T. A. (2021). Grappling with our therapeutic relationship and professional self-doubt during COVID-19: will we use video therapy again? Couns. Psychol. Q. 34, 473–484. doi: 10.1080/09515070.2020.1773404

[ref2] Alldredge C. T. Burlingame G. M. Yang C. Rosendahl J. (2021). Alliance in group therapy: a meta-analysis. Group Dyn. Theory Res. Pract. 25, 13–28. doi: 10.1037/gdn0000135

[ref3] Alonso J. Petukhova M. Vilagut G. Chatterji S. Heeringa S. Üstün T. B. . (2011). Days out of role due to common physical and mental conditions: results from the WHO world mental health surveys. Mol. Psychiatry 16, 1234–1246. doi: 10.1038/mp.2010.101, PMID: 20938433 PMC3223313

[ref4] Banbury A. Nancarrow S. Dart J. Gray L. Parkinson L. (2018). Telehealth interventions delivering home-based support group videoconferencing: systematic review. J. Med. Internet Res. 20:e25. doi: 10.2196/jmir.8090, PMID: 29396387 PMC5816261

[ref5] Batterham P. J. Calear A. L. (2017). Preferences for internet-based mental health interventions in an adult online sample: findings from an online community survey. JMIR Mental Health 4:e7722. doi: 10.2196/mental.7722 PMC5511366 28666976

[ref6] Békés V. Aafjes-van Doorn K. Luo X. Prout T. A. Hoffman L. (2021). Psychotherapists’ challenges with online therapy during COVID-19: concerns about connectedness predict therapists’ negative view of online therapy and its perceived efficacy over time. Front. Psychol. 12. doi: 10.3389/fpsyg.2021.705699, PMID: 34367030 PMC8339462

[ref7] Bipolar Commission UK (2022) Bipolar Minds Matter. Available online at: https://www.bipolaruk.org/media/filer_public/d8/ef/d8efb654-ab0e-428a-b396-19aad7452d6a/bipolar-commission-bipolar-minds-matter-november-2022.pdf

[ref8] Brainard R. Watson L. (2020). Zoom in the classroom: transforming traditional teaching to incorporate real-time distance learning in a face-to-face graduate physiology course. FASEB J. 34:1. doi: 10.1096/fasebj.2020.34.s1.08665

[ref9001] Braun V. Clarke V. (2006). Using thematic analysis in psychology. Qual. Res. Psychol. 3, 77–101. doi: 10.1191/1478088706qp063oa

[ref9] Buizza C. Candini V. Ferrari C. Ghilardi A. Saviotti F. M. Turrina C. . (2019). The long-term effectiveness of psychoeducation for bipolar disorders in mental health services. A 4-year follow-up study. Front. Psych. 10:873. doi: 10.3389/fpsyt.2019.00873, PMID: 31849726 PMC6901938

[ref10] Carlbring P. Andersson G. Cuijpers P. Riper H. Hedman-Lagerlöf E. (2018). Internet-based vs. face-to-face cognitive behavior therapy for psychiatric and somatic disorders: an updated systematic review and meta-analysis. Cogn. Behav. Ther. 47, 1–18. doi: 10.1080/16506073.2017.1401115, PMID: 29215315

[ref11] Castonguay L. G. Constantino M. J. Holtforth M. G. (2006). The working alliance: where are we and where should we go? Psychother. Theory Res. Pract. Train. 43:271. doi: 10.1037/0033-3204.43.3.271 22122096

[ref12] Chakrabarti S. Singh N. (2022). Psychotic symptoms in bipolar disorder and their impact on the illness: a systematic review. World J. Psychiatry 12, 1204–1232. doi: 10.5498/wjp.v12.i9.1204, PMID: 36186500 PMC9521535

[ref13] Chatterjee S. S. Malathesh Barikar C. Mukherjee A. (2020). Impact of COVID-19 pandemic on pre-existing mental health problems. Asian J. Psychiatr. 51:102071. doi: 10.1016/j.ajp.2020.102071, PMID: 32334407 PMC7165115

[ref14] Chiang K. J. Tsai J. C. Liu D. Lin C. H. Chiu H. L. Chou K. R. (2017). Efficacy of cognitive-behavioral therapy in patients with bipolar disorder: a meta-analysis of randomized controlled trials. PLoS One 12:e0176849. doi: 10.1371/journal.pone.0176849, PMID: 28472082 PMC5417606

[ref15] Cipolletta S. Frassoni E. Faccio E. (2018). Construing a therapeutic relationship online: an analysis of videoconference sessions. Clin. Psychol. 22, 220–229. doi: 10.1111/cp.12117

[ref16] Clemente A. S. Diniz B. S. Nicolato R. Kapczinski F. P. Soares J. C. Firmo J. O. . (2015). Bipolar disorder prevalence: a systematic review and meta-analysis of the literature. Rev. Bras. Psiquiatr. 37, 155–161. doi: 10.1590/1516-4446-2012-1693, PMID: 25946396

[ref17] Connolly S. L. Miller C. J. Lindsay J. A. Bauer M. S. (2020). A systematic review of providers’ attitudes toward telemental health via videoconferencing. Clin. Psychol. Sci. Pract. 27:e12311. doi: 10.1111/cpsp.12311 PMC9367168 35966216

[ref19] Costa R. T. D. Range B. P. Malagris L. E. N. Sardinha A. Carvalho M. R. D. Nardi A. E. (2010). Cognitive–behavioral therapy for bipolar disorder. Expert. Rev. Neurother. 10, 1089–1099. doi: 10.1586/ern.10.75, PMID: 20586690

[ref20] Czajkowski S. M. Powell L. H. Adler N. Naar-King S. Reynolds K. D. Hunter C. M. . (2015). From ideas to efficacy: the ORBIT model for developing behavioral treatments for chronic diseases. Health Psychol. 34, 971–982. doi: 10.1037/hea0000161, PMID: 25642841 PMC4522392

[ref21] Das-Munshi J. Bhugra D. Crawford M. J. (2018). Ethnic minority inequalities in access to treatments for schizophrenia and schizoaffective disorders: findings from a nationally representative cross-sectional study. BMC Med. 16:55. doi: 10.1186/s12916-018-1035-5, PMID: 29669549 PMC5904997

[ref22] De Hert M. Correll C. U. Bobes J. Cetkovich-Bakmas M. Cohen D. A. N. Asai I. . (2011). Physical illness in patients with severe mental disorders. I. Prevalence, impact of medications and disparities in health care. World Psychiatry 10, 52–77. doi: 10.1002/j.2051-5545.2011.tb00014.x, PMID: 21379357 PMC3048500

[ref23] Department of Health and Social Care (2020). Coronavirus (COVID-19). GOV.UK. Available online at: https://www.gov.uk/coronavirus?utm_campaign=coronavirus_grants&utm_medium=paid:searchl&utm_source=google&utm_content=keyword&gclid=CjwKCAjwx6WDBhBQEiwA_dP8rYN4ODPuDphOTQbAQAoLJTfkhI89ckiNUKrFSRCcNVDJxom9obIy1xoCxeQQAvD_BwE

[ref24] Dunn K. Wilson J. (2021). When online and face to face counseling work together: assessing the impact of blended or hybrid approaches, where clients move between face-to-face and online meetings. Person-Centered Exp. Psychother. 20, 312–326. doi: 10.1080/14779757.2021.1993970

[ref25] Fagiolini A. Forgione R. Maccari M. Cuomo A. Morana B. Dell’Osso M. C. . (2013). Prevalence, chronicity, burden and borders of bipolar disorder. J. Affect. Disord. 148, 161–169. doi: 10.1016/j.jad.2013.02.001, PMID: 23477848

[ref26] Fajutrao L. Locklear J. Priaulx J. Heyes A. (2009). A systematic review of the evidence of the burden of bipolar disorder in Europe. Clin. Pract. Epidemiol. Ment. Health 5, 1–8. doi: 10.1186/1745-0179-5-3 PMC2646705 19166608

[ref27] Gadelrab R. Simblett S. Hook J. Rickwood S. Martinez J. Johnstone M. . (2022). Creating a digital psychoeducation programme for bipolar disorder in the COVID-19 pandemic. Eur. Psychiatry 65:S569. doi: 10.1192/j.eurpsy.2022.1458

[ref28] Gentry M. T. Lapid M. I. Clark M. M. Rummans T. A. (2019). Evidence for telehealth group-based treatment: a systematic review. J. Telemed. Telecare 25, 327–342. doi: 10.1177/1357633X18775855, PMID: 29788807

[ref29] Gerrits R. S. van der Zanden R. A. Visscher R. F. Conijn B. P. (2007). Master your mood online: a preventive chat group intervention for adolescents. Aust. E-J. Adv. Ment. Health 6, 152–162. doi: 10.5172/jamh.6.3.152

[ref30] Grande I. Berk M. Birmaher B. Vieta E. (2016). Bipolar disorder. Lancet 387, 1561–1572. doi: 10.1016/S0140-6736(15)00241-X, PMID: 26388529

[ref31] Greene C. J. Morland L. A. Macdonald A. Frueh B. C. Grubbs K. M. Rosen C. S. (2010). How does tele-mental health affect group therapy process? Secondary analysis of a noninferiority trial. J. Consult. Clin. Psychol. 78, 746–750. doi: 10.1037/a0020158, PMID: 20873910

[ref32] Hammen C. Gitlin M. (1997). Stress reactivity in bipolar patients and its relation to a prior history of bipolar disorder. Am. J. Psychiatry 154, 856–857. 9167516 10.1176/ajp.154.6.856

[ref33] Hassija C. Gray M. J. (2011). The effectiveness and feasibility of videoconferencing technology to provide evidence-based treatment to rural domestic violence and sexual assault populations. Telemedicine e-Health 17, 309–315. doi: 10.1089/tmj.2010.0147, PMID: 21457012

[ref34] Jann M. W. (2014). Diagnosis and treatment of bipolar disorders in adults: a review of the evidence on pharmacologic treatments. American Health Drug Benefits 7:489. 25610528 PMC4296286

[ref9002] Jones S. Akers N. Eaton J. Tyler E. Gatherer A. Brabban A. (2018). Improving access to psychological therapies (IAPT) for people with bipolar disorder: Summary of outcomes from the IAPT demonstration site. Behav. Res. Ther. 111, 27–35. doi: 10.1016/j.brat.2018.09.006 30291969

[ref35] Kawa I. Carter J. D. Joyce P. R. Doughty C. J. Frampton C. M. Wells J. . (2005). Gender differences in bipolar disorder: age of onset, course, comorbidity, and symptom presentation. Bipolar Disord. 7, 119–125. doi: 10.1111/j.1399-5618.2004.00180.x, PMID: 15762852

[ref36] Kocsis B. J. Yellowlees P. (2018). Telepsychotherapy and the therapeutic relationship: principles, advantages, and case examples. Telemedicine e-Health 24, 329–334. doi: 10.1089/tmj.2017.0088, PMID: 28836902

[ref37] Lopez A. Rothberg B. Reaser E. Schwenk S. Griffin R. (2020). Therapeutic groups via video teleconferencing and the impact on group cohesion. Mhealth 6:13. doi: 10.21037/mhealth.2019.11.04, PMID: 32270005 PMC7136655

[ref38] Lustgarten S. D. Garrison Y. L. Sinnard M. T. Flynn A. W. (2020). Digital privacy in mental healthcare: current issues and recommendations for technology use. Curr. Opin. Psychol. 36, 25–31. doi: 10.1016/j.copsyc.2020.03.012, PMID: 32361651 PMC7195295

[ref9003] Malterud K. Siersma V. D. Guassora A. D. (2016). Sample Size in Qualitative Interview Studies: Guided by Information Power. Qual. Health Res. 26, 1753–1760. doi: 10.1177/1049732315617444 26613970

[ref39] Memon A. Taylor K. Mohebati L. M. Sundin J. Cooper M. Scanlon T. . (2016). Perceived barriers to accessing mental health services among black and minority ethnic (BME) communities: a qualitative study in Southeast England. BMJ Open 6:e012337. doi: 10.1136/bmjopen-2016-012337, PMID: 27852712 PMC5128839

[ref40] Merikangas K. R. Jin R. He J. P. Kessler R. C. Lee S. Sampson N. A. . (2011). Prevalence and correlates of bipolar spectrum disorder in the world mental health survey initiative. Arch. Gen. Psychiatry 68, 241–251. doi: 10.1001/archgenpsychiatry.2011.12, PMID: 21383262 PMC3486639

[ref41] Morland L. A. Hynes A. K. Mackintosh M. A. Resick P. A. Chard K. M. (2011). Group cognitive processing therapy delivered to veterans via telehealth: a pilot cohort. J. Trauma. Stress. 24, 465–469. doi: 10.1002/jts.20661, PMID: 21793047

[ref42] Müller-Oerlinghausen B. Berghöfer A. Bauer M. (2002). Bipolar disorder. Lancet 359, 241–247. doi: 10.1016/S0140-6736(02)07450-0, PMID: 11812578

[ref43] Naslund J. A. Aschbrenner K. A. Araya R. Marsch L. A. Unützer J. Patel V. . (2017). Digital technology for treating and preventing mental disorders in low-income and middle-income countries: a narrative review of the literature. Lancet Psychiatry 4, 486–500. doi: 10.1016/S2215-0366(17)30096-2, PMID: 28433615 PMC5523650

[ref44] National Health Service. (2019). NHS mental health implementation plan 2019/20 – 2023/24. Available online at: https://www.longtermplan.nhs.uk/wp-content/uploads/2019/07/nhs-mental-health-implementation-plan-2019-20-2023-24.pdf; https://www.england.nhs.uk/wp-content/uploads/2022/07/nhs-mental-health-implementation-plan-2019-20-2023-24.pdf

[ref45] National Institute for Health and Care Excellence. (2014). Bipolar disorder: assessment and management [clinical guideline CG185]. https://www.nice.org.uk/guidance/cg185 31487127

[ref46] Naveed S. Waqas A. Chaudhary A. M. D. Kumar S. Abbas N. Amin R. . (2020). Prevalence of common mental disorders in South Asia: a systematic review and meta-regression analysis. Front. Psych. 11:573150. doi: 10.3389/fpsyt.2020.573150, PMID: 32982812 PMC7492672

[ref47] Newton E. Matharu G. Jones C. A. Kaufman A. Yagnik R. McDonald S. . (2025). The feasibility and acceptability of mood on track: an online psychological intervention for bipolar disorder. Int. J. Bipolar Disord. 13:22. doi: 10.1186/s40345-025-00385-8, PMID: 40490645 PMC12149070

[ref48] Novick D. M. Swartz H. A. Frank E. (2010). Suicide attempts in bipolar I and bipolar II disorder: a review and meta-analysis of the evidence. Bipolar Disord. 12, 1–9. doi: 10.1111/j.1399-5618.2009.00786.x, PMID: 20148862 PMC4536929

[ref49] Perich T. Kakakios K. Fraser I. (2023). ‘I almost felt like I can be a little bit more honest’: experiences of a telehealth group for bipolar disorder. Cogn. Behav. Ther. 16:e26. doi: 10.1017/S1754470X2300020X

[ref50] Perich T. Kakakios K. Fraser I. (2024). Telehealth-delivered recovery-orientated well-being plan group program for bipolar disorder: a pilot randomised feasibility and acceptability study. Behav. Cogn. Psychother. 52, 681–686. doi: 10.1017/S1352465824000316, PMID: 39257346

[ref51] Perra A. Sancassiani F. Cantone E. Pintus E. D’Oca S. Casula A. . (2024). An e-health psychoeducation program for managing the mental health of people with bipolar disorder during the COVID-19 pandemic: a randomized controlled study. J. Clin. Med. 13:3468. doi: 10.3390/jcm13123468 38929997 PMC11204713

[ref52] Pini S. Abelli M. Mauri M. Muti M. Iazzetta P. Banti S. . (2005). Clinical correlates and significance of separation anxiety in patients with bipolar disorder. Bipolar Disord. 7, 370–376. doi: 10.1111/j.1399-5618.2005.00216.x, PMID: 16026490

[ref53] Rafieifar M. Hanbidge A. S. Lorenzini S. B. Macgowan M. J. (2025). Comparative efficacy of online vs. face-to-face group interventions: a systematic review. Res. Soc. Work. Pract. 35, 524–542. doi: 10.1177/10497315241236966

[ref54] Rees C. S. Stone S. (2005). Therapeutic alliance in face-to-face versus videoconferenced psychotherapy. Prof. Psychol. Res. Pract. 36, 649–653. doi: 10.1037/0735-7028.36.6.649

[ref55] Reynolds D. A. J. Jr. Stiles W. B. Bailer A. J. Hughes M. R. (2013). Impact of exchanges and client–therapist alliance in online-text psychotherapy. Cyberpsychol. Behav. Soc. Netw. 16, 370–377. doi: 10.1089/cyber.2012.0195 23530546 PMC3677235

[ref56] Sansom-Daly U. M. Wakefield C. E. Bryant R. A. Patterson P. Anazodo A. Butow P. . (2019). Feasibility, acceptability, and safety of the recapture life videoconferencing intervention for adolescent and young adult cancer survivors. Psycho-Oncology 28, 284–292. doi: 10.1002/pon.4938, PMID: 30414219

[ref57] Spanakis P. Wadman R. Walker L. Heron P. Mathers A. Baker J. . (2024). Measuring the digital divide among people with severe mental ill health using the essential digital skills framework. Perspect. Public Health 144, 21–30. doi: 10.1177/17579139221106399, PMID: 35929589 PMC10757390

[ref58] Stoll J. Müller J. A. Trachsel M. (2020). Ethical issues in online psychotherapy: a narrative review. Front. Psych. 10:993. doi: 10.3389/fpsyt.2019.00993, PMID: 32116819 PMC7026245

[ref59] Sucala M. Schnur J. B. Constantino M. J. Miller S. J. Brackman E. H. Montgomery G. H. (2012). The therapeutic relationship in e-therapy for mental health: a systematic review. J. Med. Internet Res. 14:e2084. doi: 10.2196/jmir.2084 PMC3411180 22858538

[ref60] Watson S. Dodd A. (2017). Structured group psychoeducation in patients with bipolar disorder delays time to mania and time to any episode compared with a peer support group. Evid. Based Ment. Health 20:e15. doi: 10.1136/eb-2017-102648, PMID: 28689180 PMC10688552

[ref61] Wentzel J. van der Vaart R. Bohlmeijer E. T. van Gemert-Pijnen J. E. (2016). Mixing online and face-to-face therapy: how to benefit from blended care in mental health care. JMIR Mental Health 3:e4534. doi: 10.2196/mental.4534 PMC4764785 26860537

[ref62] Xue S. Husain M. I. Ortiz A. Husain M. O. Daskalakis Z. J. Mulsant B. H. (2020). COVID-19: implications for bipolar disorder clinical care and research. SAGE Open Medicine 8:2050312120981178. doi: 10.1177/2050312120981178, PMID: 33403113 PMC7739076

[ref63] Zhang L. Cao X. L. Wang S. B. Zheng W. Ungvari G. S. Ng C. H. . (2017). The prevalence of bipolar disorder in China: a meta-analysis. J. Affect. Disord. 207, 413–421. doi: 10.1016/j.jad.2016.08.062, PMID: 27771597

